# Antiproliferative and Pro-Apoptotic Effects of MiR-4286 Inhibition in Melanoma Cells

**DOI:** 10.1371/journal.pone.0168229

**Published:** 2016-12-22

**Authors:** Anna Komina, Nadezhda Palkina, Mariya Aksenenko, Seseg Tsyrenzhapova, Tatiana Ruksha

**Affiliations:** Department of Pathophysiology, Krasnoyarsk State Medical University, Krasnoyarsk, Russian Federation; Universita degli Studi di Napoli Federico II, ITALY

## Abstract

**Introduction:**

MicroRNAs are essential regulators of gene expression at the post-transcriptional level. Their expression is altered in cancer tissues, and evaluation of these alterations is considered a promising tool used to diagnose and identify prognostic markers.

**Materials and methods:**

The microRNA expression profiles of formalin-fixed, paraffin-embedded melanoma and melanocytic nevi samples were estimated with a microarray and subsequently validated by real-time PCR. Melanoma cells were transfected with miR-4286 inhibitor to evaluate the influence of this microRNA on the viability, proliferation, apoptosis, migration, and invasion of melanoma cells.

**Results:**

The microarray revealed that the expression of 1,171 microRNAs was altered in melanoma samples compared to melanocytic nevi. Real-time PCR validation experiments found the microRNA expression levels to correspond to the melanoma/melanocytic nevi microarray results. The pathway analysis identified 52 modulated pathways in melanoma. Moreover, the application of miR-4286 inhibitor to BRO melanoma cells resulted in a 2.6-fold increase in the apoptosis rate and a 1.7-fold decrease in the cell proliferation/viability but did not affect the invasiveness and migration of these cells. Furthermore, the use of miR-4286 inhibitor altered the mRNA expression of several miR-4286 gene targets: folylpolyglutamate synthase, RNA polymerase I-specific transcription initiation factor, apelin, G-protein-coupled receptor 55, and high-mobility group A1 protein, which have been implicated in cell proliferation/apoptosis regulation. Lastly, the transiently transfected SK-MEL-1 cells with miR-4286 inhibitor decreased proliferation rate and modulated folylpolyglutamate synthase rates of these cells.

**Conclusion:**

Our results demonstrate that miR-4286 mediates proliferation and apoptosis in melanoma cells, these findings may represent a novel mechanism underlying these processes.

## Introduction

MicroRNAs are small (19–24 nucleotides), non-coding RNA molecules that regulate gene expression at the post-transcriptional level and hundreds of microRNAs have been discovered and investigated to date [1]. A single microRNA can regulate the expression of more than 100 genes. In turn, a gene may be targeted by several microRNAs. Given the large number of miRNAs annotated in the human genome, 30%–80% of human genes are predicted to be regulated by miRNAs [2; 3; 4; 5; 6; 7]. Specifically, aberrant microRNA profiles have been observed in various cancer types, which may be a result of epigenetic alterations of miRNA promoters, the altered activity of transcriptional factors or dysregulated enzymes involved in miRNA biogenesis [8]. Furthermore, altered microRNA expression contributes to cancer development by down-regulating tumor suppressor genes or up-regulating tumor-promoting oncogenes [9].

Melanoma is an aggressive skin cancer whose incidence rate has been increasing within white-skinned populations [10]. The survival of patients with melanoma depends on tumor thickness, which is one of the most important prognostic factors. Therefore, the early detection of the tumor is crucial for a successful treatment, and the detection of changes in the microRNA profile in tumor tissue may serve as a tool for identification of novel molecular markers. Recent studies showed that melanoma progression is accompanied by significant dysregulation of the microRNA expression pattern [11, 12], but a specific microRNA has not yet been shown as an adequate marker to diagnose and predict the prognosis of patients with this disease. This challenge may be due to tumor heterogeneity, which complicates the differentiation of microRNA expression pattern between tumor and tumor microenvironment cells. Conversely, the tissue microRNA expression pattern is relatively stable and specific under different pathological states. Specifically, a recent study [13] identified the following five microRNAs to be strongly associated with the outcome of patients with melanoma: miR-142-5p, miR-150-5p, miR-342-3p, miR-155-5p, and miR-146b-5p. Unfortunately, the serum-derived microRNAs of these patients correlate only with the microRNA tissue pattern at the late stage but not with primary tumors which currently complicates the use of microRNA identification as prognostic or diagnostic approach for melanoma [14]. Thus, additional studies are necessary to clarify the exact role of altered microRNAs in tumor development and progression as well as their clinical importance. MiR-4286 was shown to be up-regulated in different types of malignancies such as pancreatic cancer [15], glioma [16], and esophageal adenocarcinoma [17]. Furthermore, Sand M. et al. included miR-4286 in a list of significantly up-regulated microRNAs in melanoma whose role in melanomagenesis has not been studied [18].

The aim of this study was to compare the microRNA expression profiles in melanoma and melanocytic nevi in order to identify a specific pattern and study the function of aberrantly expressed microRNAs in melanoma.

## Materials and Methods

### Tissue samples

The study was approved by the Krasnoyarsk State Medical University Local Ethics Committee (protocol №59/2014 issued on December 2, 2014). Written consent was approved by the Local Ethics Committee and obtained from all participants in the study before obtaining samples. Treatment-naive primary melanoma and benign melanocytic tumor samples were obtained from patients at the Krasnoyarsk Regional Oncology Center, which is named after A.I. Kryzhanovsky. Sixteen melanoma and 3 melanocytic nevi samples were fixed in formalin and embedded in paraffin. The ages of patients with melanoma ranged from 35 to 81 years. Men comprised 56% and females– 44% of the cohort (Table 1). The ages of patients with melanocytic nevi ranged from 29 to 55 years. Two patients had nevi on the face, whereas one was with nevus on the breast (Table 2). Samples of melanocytic lesions containing less than 70% of studied cells were macrodissected to separate the tissues from melanoma cells and tumor microenvironment. The percentage of target tissue in each sample was evaluated by hematoxylin/eosin staining and subsequent light microscopy.

**Table 1:** Clinical and demographic details of patients with melanoma included in the study.

Patient №	Gender	Age	Clinical type	Anatomic location	Breslow thickness, mm	Clark's level
**1**	Male	46	Acral lentiginous	Palm	5.80	V
**2**	Female	41	Superficial spread	Head	3.20	III
**3**	Female	75	Superficial spread	Shoulder	4.00	IV
**4**	Male	50	Acral lentiginous	Foot	4.00	IV
**5**	Female	62	Superficial spread	Back	No data	IV
**6**	Female	36	Superficial spread	Back	3.62	IV
**7**	Female	35	Superficial spread	Thigh	4.46	IV
**8**	Male	63	Superficial spread	Back	No data	III
**9**	Female	51	Superficial spread	Abdomen	4.27	IV
**10**	Male	50	Superficial spread	Forearm	5.80	V
**11**	Male	66	Nodular	Back	7.07	V
**12**	Male	63	Acral lentiginous	Foot	3.00	IV
**13**	Male	56	Acral lentiginous	Palm	6.85	IV
**14**	Female	81	No data	Shoulder	5.00	IV-V
**15**	Male	73	Nodular	Shoulder	2.00	III
**16**	Male	62	Nodular	Back	3.00	III-IV

**Table 2:** Characteristics of patients with melanocytic nevi.

Patient №	Gender	Age	Anatomic location	Diagnosis
**1**	Female	29	Head	Compound melanocytic nevus *with papillomatous features*
**2**	Female	55	Breast	Intradermal melanocytic nevus *with papillomatous features*
**3**	Female	34	Head	Compound melanocytic nevus *with papillomatous features*

Samples were processed according to a standard protocol that included incubation in 10% neutral buffered formalin for 24 h and embedding in paraffin blocks. The diagnosis was made by a certified pathologist at the Krasnoyarsk Pathologic Anatomy Bureau. After fixation, the samples were stored in the Archive of Pathologic Anatomy Bureau at room temperature for up to 5 years.

### RNA isolation

Total RNA was isolated using the RecoverAll™ Total Nucleic Acid Isolation kit (Ambion, Life Technologies, Vilnius, Lithuania). Formalin-fixed and paraffin-embedded (FFPE) samples were sectioned at a thickness of 15 μm, deparaffinized with xylene, and washed twice with ethanol according to the standard procedure. The tissues were then suspended in 200 μl of Digestion buffer. RNA was isolated according to manufacturer’s instructions for RNA isolation and eluted with 50 μl of nuclease-free water. The microRNA concentration was measured on Qubit® 2.0 fluorimeter (Invitrogen by Life Technologies, Singapore, Singapore) using the Qubit® microRNA Assay kit (Ref. Q32880, Eugene, Oregon, USA). The concentration of microRNA in the samples submitted to the microarray analysis exceeded 16.3 ng/μl.

### Microarray

The microarray analysis was conducted on a GeneAtlas™ Microarray System (Affymetrix, Santa Clara, CA, USA). The total RNA samples containing microRNA concentrations ranging from 16.3 to 40.8 ng/μl were labeled with biotin using Affymetrix® Flash Tag™ Biotin HSR (Ref. 901913, Affymetrix, Santa Clara, CA, USA). The labeled molecules were then hybridized at 48°C for 20 h on arrays in Affymetrix® miRNA 4.1 Array Strip (Affymetrix, Santa Clara, USA) using the GeneChip® GeneAtlas™ Hybridization and Stain Module (Ref. 902135) reagents (Affymetrix, Santa Clara, CA, USA). After hybridization, the arrays were washed on the GeneAtlas™ Microarray System Fluidic Station using the GeneChip® GeneAtlas™ Hybridization and Stain Module buffers and solutions (Ref. 902135) (Affymetrix, Santa Clara, CA, USA). The intensities of the fluorescent signal were detected on the Imaging Station of the GeneAtlas™ Microarray System. Both spike control oligos and hybridization control stages of the procedure were performed according to the manufacturer’s instructions and under quality control. The Quality Control (QC) of the experiment was automatically carried out at the imaging stage, and only samples that passed the QC threshold were used for the following analysis. The microarray data have been deposited in a MIAME-compliant format [19] to the public data repository ArrayExpress with accession number E-MTAB-4915.

### TaqMan miRNA real-time PCR

In this work, the Minimum Information for Publication of Quantitative Real-Time PCR Experiments (MIQE) guidelines was applied for data reporting [20]. The reverse transcription reaction was performed in a reaction mixture containing 0.4 μl of 5x primers miRNA TaqMan assays specific to the investigated microRNA (Cat. № 4427975, Applied Biosystems, Foster City, CA, USA) or Random-primers for mRNA expression analysis, 0.4 μl of OT-buffer, 0.05 μl revertase enzyme, and 0.15μl of RNA-eluent solutions from the “Reverta” kit (AmpliSens, Moscow, Russia) per 1 μl of isolated RNA. The reactions were carried out at 37°C for 30 min. cDNA was immediately used for the RT-PCR reaction or stored at -20°C. Briefly, the obtained cDNA was added to a PCR cocktail containing 8 μl of the 2.5-fold RT-PCR reaction mixture and ROX reference dye (Syntol, Moscow, Russia), 1 μl of 20x primers, miRNA TaqMan assay probe mix, and deionized water to a total volume of 20μl. U6snRNA and RNU6B (Cat. № 001973, Assay ID 001973 and 4427975 respectively, Applied Biosystems, Foster City, CA, USA) were used as endogenous controls to estimate the relative microRNA expression levels; beta-actin (Cat. № 4331182, Assay ID Hs01060665_g1) and HPRT-1 (hypoxanthinephosphoribosyltransferase 1) (Cat. № 4331182, Assay ID Hs01003267_m1) were used as endogenous controls to evaluate mRNA levels. The reaction was performed on a StepOne™ Real-Time PCR System (Applied Biosystems, Singapore, Singapore) with the following temperature cycling protocol: 50°C for 2 min and 95°C for 10 min, followed by 40 cycles of 95°C for 15 sec and 60°C for 1 min with FAM/VIC detection. Three replicates were performed for each experiment. The microRNA or mRNA expression was normalized to the geometric mean of U6 snRNA and RNU6B or beta-actin and HPRT-1, respectively, as described previously [21]. The data were analyzed using the ΔΔC_t_ method.

### Cell culture and transfection

The human melanoma cell line BRO [22; 23] was obtained from the Research Institute of Fundamental and Clinical Immunology (Novosibirsk, Russian Federation). SK-MEL-1 cells [24, 25] were a gift from the A.N. Sysin Research Institute of Human Ecology and Environmental Health at the Ministry of Health of the Russian Federation (Moscow, Russian Federation). The cells were grown in RPMI-1640 medium containing L-glutamine (Gibco, Life Technologies, Paisley, UK) supplemented with 10% fetal bovine serum (Gibco, Life Technologies, Paisley, UK) at 37°C in Sanyo MSO-5AC CO_2_ incubator (Sanyo Electric Co., Ltd., Osaka, Japan) containing 5% CO_2_.

The 50 mM anti-miR water solution was diluted in 500 μl of cell-containing culture medium to a final concentration of 25 nM. The medium was replaced every 24 h. To evaluate the transfection efficiency, anti-miR^TM^ hsa-let-7c miRNA inhibitor (Cat. № 4392431, Ambion, Carlsbad, CA, USA) and anti-miR^TM^ negative control *#*1 (Cat. № AM17010, Ambion, Carlsbad, CA, USA) were transfected into Bro and SK-MEL-1 melanoma cells in a 24-well plate in concentration of 1×10^5^ cells/ml using 1.5 μl Lipofectamine 2000 reagent (Invitrogen, Life Technologies, Carlsbad, CA, USA) per well. The transfection efficiency was estimated by detecting changes in the expression of high-mobility group AT-hook 2 (HMGA2, Assay ID Hs00171569_m1, Cat № 4331182, Applied Biosystems, Foster City, CA, USA) in the transfected cells using real-time PCR detection. Because hsa-let-7c specifically down-regulates the expression of HMGA2, the effective inhibition of let-7c increases the HMGA2 mRNA level in cells. Moreover, the miR-4286 expression levels were evaluated in both melanoma cell lines after transfection with hsa-miR-4286 anti-miR^TM^ miRNA inhibitor (Cat. № AM17000, Assay ID AM 17048, Ambion, Life Technologies, Carlsbad, CA, USA) using real-time PCR and 5X specific from primers of TaqMan MicroRNA Assay (Cat. № 4427975, Assay ID 241500_mat, Applied Biosystems, Foster City, CA, USA), as described above. The experiments were performed in triplicate.

### Cell apoptosis detection

#### Flow cytometric analysis of cell apoptosis

Cells transfected with hsa-miR-4286 anti-miR^TM^ miRNA inhibitor (Cat. № AM17000, Ambion, Life Technologies, Carlsbad, CA, USA) or with anti-miR^TM^ negative control *#*1 (Cat. № AM17010, Ambion, Life Technologies, Carlsbad, USA) were cultured in 24-well plates at 37°C in 5% CO_2_. Forty-eight hours after transfection, the cells were detached with 0.25% trypsin in HBSS (Gibco®, Paisley, UK), washed twice in cold PBS, and suspended in 100 μl of 1X binding buffer from the Annexin V–FITC/7AAD kit (Immunotech, BeckmanCoulter, Marseille, France). Subsequently, 1 μl of Annexin V–FITC and 20 μl 7AAD were added and the samples were incubated for 15 min on ice in the dark. After incubation, 400 μl 1x binding buffer was added and the proportions of viable (Annexin V-/7AAD-), early apoptotic (Annexin V+/7AAD-), late apoptotic/necrotic (Annexin V+/7AAD+), and necrotic (Annexin V-/7AAD+) cells were detected on a Cytomics FC-500 instrument (BeckmanCoulter, Brea, CA, USA). The experiment was performed in triplicate.

#### Fluorescent microscopy

Cells transfected with hsa-miR-4286 anti-miR^TM^ miRNA inhibitor (Cat. № AM17000, Ambion, Life Technologies, Carlsbad, CA, USA) or anti-miR^TM^ negative control *#*1 (Cat. № AM17010, Ambion, Life Technologies, Carlsbad, CA, USA) were cultured in 100 μl of RPMI-1640 medium at a final concentration of 10^5^ cells/ml in 96-well plates. The cells were incubated at 37°C in 5% CO_2_. After 48-h incubation, fresh RPMI medium containing L-glutamine and 10% FBS was mixed with NucBlue® Live fluorescent reagents from the Ready Probes® Cell Viability Imaging Kit (Blue/Red) (Molecular probes, Life Technologies, Eugene, Oregon, USA) and CellEvent® Caspase-3/7 Green (Molecular probes, Life Technologies, Eugene, Oregon, USA) according to the manufacturer’s protocol. The culture medium in the wells was then replaced with a medium containing both fluorescent reagents mentioned above according to the manufacturer’s protocol. After 30 min of incubation, cells positive for Caspase-3/7 were visualized and photographed using Floid® Cell Imaging Station (Life Technologies, Bothell, WA, USA). The nuclei of living cells were stained in blue by NucBlue® Live reagent (Molecular probes, Life Technologies, Eugene, Oregon, USA). The cytoplasm of Caspases-3/7 positive cells appeared green.

### Cell viability/proliferation MTT-test

Cells transfected with hsa-miR-4286 anti-miR^TM^ miRNA inhibitor (Cat. № AM17000, Ambion, Life Technologies, Carlsbad, USA) or anti-miR^TM^ negative control *#*1 (Cat. № AM17010, Ambion, Life Technologies, Carlsbad, CA, USA) were cultured for 24 h, detached with 0.25% trypsin solution in HBSS (Gibco®, Paisley, UK), and replaced in 96-well plates in a final medium volume of 100 μl per well and a concentration of 3∙10^4^ cells per ml. MTT assays were performed 24, 48, 72, and 96 h after transfection. To this end, the culture medium was replaced and 10 μl of 2-(4,5-dimethyltriazol2yl)-2,5-diphenyltetrazolium bromide (MTT) solution was added to each well. The cells were incubated with MTT for 4 h at 37°C in 5% CO_2_. The solution was then removed, followed by dissolving the pellet with 200 μl of DMSO per well before being incubated at room temperature for 30 min. The absorbance of stained supernatants was measured with an EFOS-9305 spectrophotometer (Shvabe-photosystems, Moscow, Russia) at 560 nm. Cell viability/proliferation was directly proportional to the absorbance. The relative cellular growth was assessed based on the ratio of average absorbance of hsa-miR-4286 anti-miR^TM^ miRNA inhibitor cells to the average absorbance of anti-miR^TM^ negative control *#*1 cells. The experiment was performed in triplicate.

### Migration and invasion assay

Migration and invasion experiments were conducted separately using a CytoSelect™ 24-Well Cell Migration and Invasion Assay (8 μm, Colorimetric Format) (Cell Biolabs, Inc., San Diego, CA, USA) and 24-well migration and invasion plates containing polycarbonate membrane inserts with 8 μm pores. The upper membrane surfaces of the inserts for the invasion assay were covered in a uniform layer of dried basement membrane matrix solution. Melanoma cells transfected with hsa-miR-4286 anti-miR^TM^ miRNA inhibitor (Cat. № AM17000, Ambion, Life Technologies, Carlsbad, CA, USA) or anti-miR^TM^ negative control *#*1 (Cat. № AM17010, Ambion, Life Technologies, Carlsbad, CA, USA) were detached with 0.25% trypsin in HBSS (Gibco®, Paisley, UK) after 24 h of transfection, washed with PBS and suspended in FBS medium RPMI-1640 free to final concentration of 1 × 10^5^ cells per ml. The cell suspension was then placed inside each insert and the bottom well of the migration plate was filled with the medium containing 10% FBS. After 22 h of incubation at 37°C in 5% CO_2_, the cells on the interior of the inserts were mechanically removed, and the migrated cells were fixed and stained with the kit’s Cell Stain Solution and dried according to manufacturer’s instructions. The stained cells were analyzed on a Floid® Cell Imaging Station (Life Technologies, Bothell, WA, USA) in three random fields. The cells were dissolved using the kit’s Extraction Solution for 10 min and transferred to 96-well plates to evaluate the optical density at 560 nm using EFOS-9305 spectrophotometer (Shvabe-photosystems, Moscow, Russia). The relative migration/invasion activity was determined by calculating the ratio of the average absorbance in hsa-miR-4286 anti-miR^TM^ miRNA inhibitor cells to the average absorbance of anti-miR^TM^ negative control *#*1 cells. The experiments were performed in triplicate.

### KEGG pathway analysis of miRNA target genes

DIANA-mirPath v.3.0 was used for the KEEG pathway analysis of the miRNA signature. MiRNA targets were predicted based on DIANA-microT-CDS and heatmaps were generated using the log-transformed enrichment p-values as features for each miRNA. The threshold (P-value ≤0.05 and false discovery rate (FDR) ≤0.05) was calculated using Fisher's exact test.

To predict the gene targets of hsa-miR-4286, three different algorithms, TargetScan 7.0, miRWalk 2.0, and miRTarBase v.4.5 were applied to identify the validated targets of hsa-miR-4286. To reduce the false-positive results, only genes identified by all three methods were selected as miRNA targets to be subsequently analyzed. Cumulative context score was applied to choose most targeted genes for miR-4286. The PANTHER™ v.10.0 classification system was used to interpret the biological function of the validated targets of hsa-miR-4286.

### Statistical analysis

The Expression Console and Transcriptome Analysis Console 2.0 (Affymetrix, USA) software were used for quality control, statistical analysis and miRNA annotation. The data were automatically statistically analyzed using ANOVA test and FDR-corrected values. MicroRNAs expression differences were assessed using a Mann-Whitney *U*-test and considered significant at p ≤0.05. The hierarchical clustering of differently expressed microRNAs was generated by the Transcriptome Analysis Console 2.0 software (Affymetrix, Santa Clara, CA, USA). Real-time PCR data were analyzed by using the ΔΔC_t_ method and a nonparametric Mann-Whitney *U*-test with the Statistica 6.1 software (StatSoft, Moscow, Russia). The data on cell proliferation, viability, apoptosis, migration, and invasion were analyzed using a nonparametric Mann-Whitney *U*-test. The differences were considered significant at p < 0.05.

## Results

### MicroRNA expression in melanoma and melanocytic nevi from FFPE tissues

**Fig 1:**
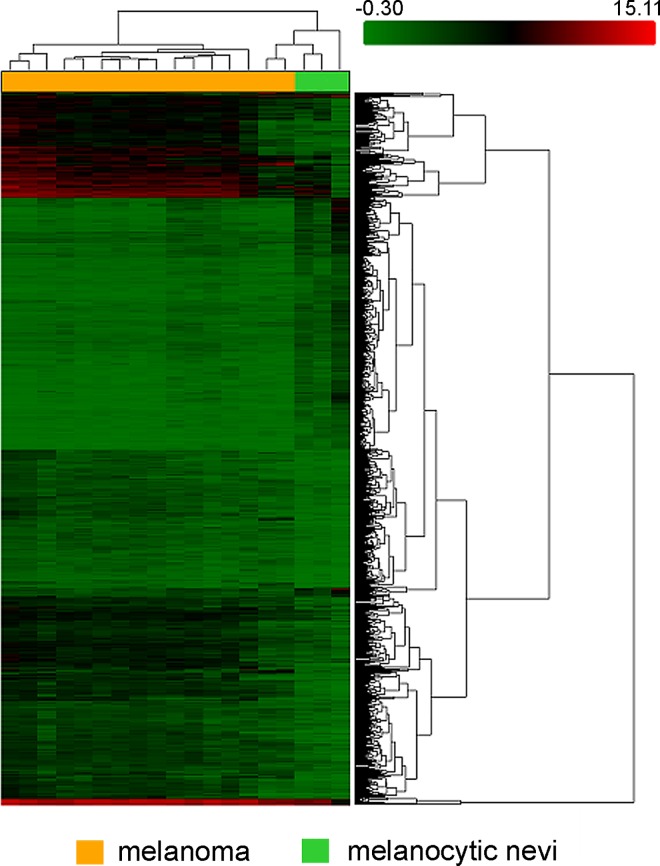
Unsupervised hierarchical clustering analysis of differentially expressed microRNAs between melanoma and melanocytic nevi samples. The heat map colors reflect the expression levels of microRNA according to a color key (from green to red).

A microarray analysis of FFPE-embedded samples identified 1,600 of 6,631 short non-coding RNAs that exhibited an at least 2-fold difference in expression between melanoma and melanocytic nevi samples. Of these RNAs, 1,171 were classified as microRNAs. Specifically, 674 microRNAs were up-regulated and 497 –down-regulated in melanoma (S1 Table). The largest differences were observed in miR-155-5p, miR-1246, let-7i-5p, miR-34a-5p, miR-652-3p, and miR-4530, which were up-regulated, and in miR-211-5p, miR-5196-3p, miR-6804-3p, miR-6809-3p, and miR-6736-3p, which were down-regulated. The hierarchical clustering clearly differentiated between the melanoma and nevus groups based on miRNA expression profiles (Fig 1).

### Real-time PCR

**Fig 2:**
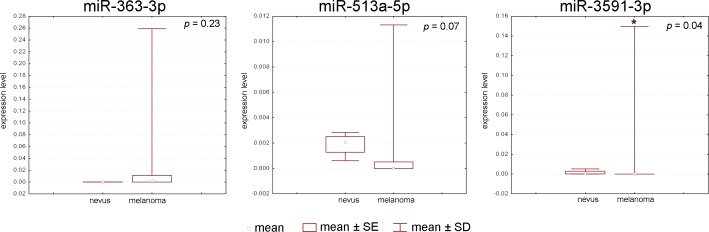
Real-time PCR verification of microRNA expression data obtained by microarray. miR-363-3p, miR-513a-5p, and miR-3591-3p were investigated. The microarray data for all three microRNAs were confirmed by real-time PCR.

The miR-363-3p, miR-513a-5p, and miR-3591-3p expression levels in melanoma and melanocytic nevi samples were measured by real-time PCR to confirm the microarray data. The MiR-513a-5p, miR-363-3p, and miR-3591-3p were selected for this analysis because they exhibited a wide range in expression differences between melanoma and melanocytic nevi samples: a preliminary microarray showed that miR-363-3p was 8.65-fold over-expressed, miR-3591-3p was 16.18-fold down-regulated and miR-513a-5p expression did not significantly differ between melanoma and nevi samples. Moreover, after adjusting p-values with FDR, the significance of miR-363-3p changes was lost, what required confirmation by real-time PCR. Furthermore, these molecules were of different size (18, 22, and 23 nucleotides for miR-513a-5p, miR-363-3p, and miR-3591-3p, respectively) and exhibited GC contents of 34.78% (miR-3591-3p), 40.91% (miR-363-3p), and 50.00% (miR-513a-5p). Moreover, previous studies indicated that matched microRNAs are involved in carcinogenesis [26, 27, 28]. Real-time PCR confirmed the microarray data obtained after FDR correction: miR-363-3p and miR-513a-5p expression did not significantly differ between melanoma and melanocytic nevi, whereas miR-3591-3p was down-regulated in melanoma compared to melanocytic nevi (Fig 2, S2 Table).

**Fig 3:**
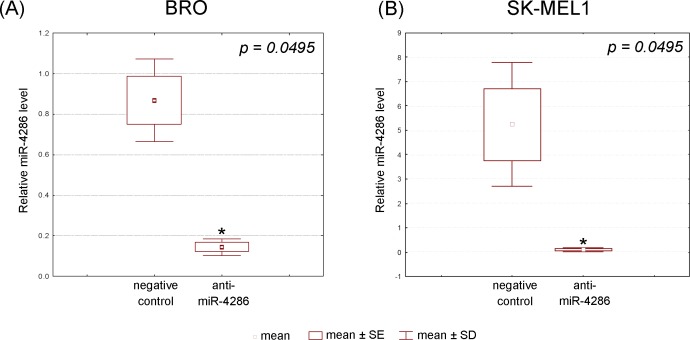
Expression levels of miR-4286 in BRO and SK-MEL-1 melanoma cells estimated by real-time PCR after miR-4286 inhibitor application (normalized by the geometric mean of U6snRNA and RNU6B expression levels).

The efficiency of cell transfection with anti-miR-4286 was estimated based on a real-time PCR analysis of HMGA2 expression in the transfected cells. Specifically, transfected BRO and SK-MEL-1 cells with let-7c miRNA inhibitor resulted in a 30% increase in HMGA2 expression compared to the negative control (p = 0.0495), (Table 3). Furthermore, real-time PCR determined that the miR-4286 levels were down-regulated in both anti-miR-4286 transfected cell types compared to the negative control (p = 0.0495), (data are presented in Fig 3, S3 Table).

**Table 3:** Expression levels of HMGA2 by PCR real-time as indicators of transfection efficiency (normalized by the geometric mean of β-actin and HPRT-1 expression levels).

Cell line	Relative quantity, mean ± SEM		P
	Negative control	Positive control	
**BRO**	1.088134±0.090764	1.425785±0.101397	0.0495
**SK-MEL1**	0.911768±0.021595	1.248513±0.146978	0.0495

### Signal pathway analysis

The study of FFPE-embedded tissues identified 52 signaling pathways as significantly modulated. Specifically, fatty acid biosynthesis, elongation, metabolism, AMPK signaling, actin cytoskeleton regulation, and melanoma pathways were altered in melanoma compared to benign melanocytic lesions.

Furthermore, melanoma samples exhibited alterations in the Hippo, viral carcinogenesis, TGF-beta signaling, cell cycle and adherens junction pathways. The full list of signaling pathways associated with differently expressed microRNAs is presented in S4 Table.

### MiR-4286

Furthermore, miR-4286 was more than 17.8-fold up-regulated in melanoma compared to benign melanocytic tumors in our study. Thus, we carried out a biometrical analysis of target genes of miR-4286 and a functional study of its activity using a miR-4286 specific inhibitor that was transfected in melanoma cells.

### Gene target analysis for miR-4286

The three algorithms (as mentioned in Materials and methods section) predicted 94 as the targets of hsa-miR-4286. Our bioinformatic studies have suggested that hsa-miR-4286 regulates a large number of target genes that are important for the regulation of crucial cell processes: cell proliferation (APLN, FPGS, FTSJ2, FURIN, HMGA1, RAPGEF3, RRN3, RPS6, and TP53); angiogenesis (PIK3C2B, MAPK1 KSR1, and RAPGEF3); cell fate, cell survival, metabolism, cell death, cell division, and mitosis (PIK3C2B, MAPK1, REL, TP53, FBXO18, RABGAP1, SLFN5, TMEM109, TRAF3, and ZBTB7A); glucose, lipids, nucleic acids, and amino acids metabolism, as well as ion transport (ACBD7, APOL6, ATP1A3, ATP13A4, CACNA1E, LDLR, MAN1A2, PGK1, PRKAG1, RIMS3, SCNN1G, SLC39A13, SLC6A17, TMEM151B, and TRPC4AP); and adhesion molecules (CLDN1, SEMA4D, and SIRPA). S5 Table shows the complete list of biological functions of miR-4286 target genes.

#### Cell viability/proliferation assay

**Fig 4:**
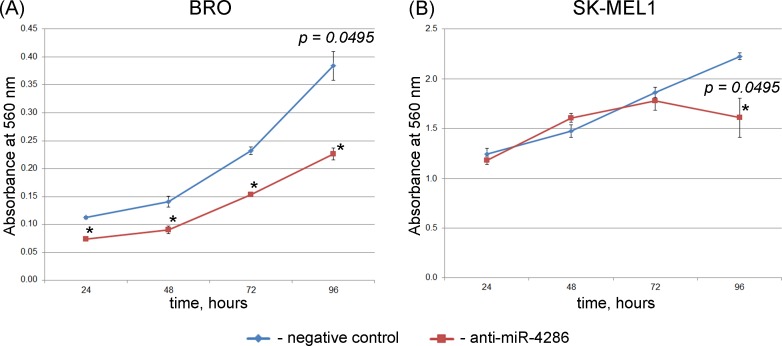
**Cell viability/proliferation (MTT assay) of BRO (A) and SK-MEL-1 (B) melanoma cells after miR-4286 inhibition.** Viability/cell proliferation is considered directly proportional to the absorbance. Data are presented as the mean ± SEM. *—significant differences compared to the negative control at the corresponding time points.

An MTT assay was performed 24, 48, 72, and 96 h after transfecting the cells with the miR-4286 inhibitor. As Fig 4 suggests, the inhibition of miR-4286 activity significantly decreased the viability/proliferation of BRO melanoma cells at each time point, whereas SK-MEL-1 cell proliferation was decreased only at the 96-h time point (Fig 4, S6 Table).

#### Apoptosis detection

**Fig 5:**
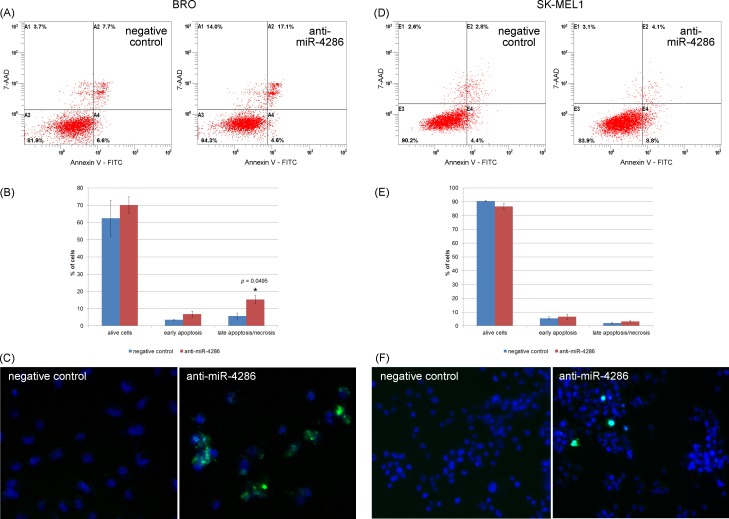
Effect of anti-miR-4286 on melanoma cell apoptosis. (A) Flow cytometry showed a significant increase in apoptotic BRO melanoma cells after miR-4286 inhibition. (B) The percentage of viable, early apoptotic, late apoptotic/necrotic BRO cells. (C) Fluorescent microscopy of alive and apoptotic BRO cells: viable cell nuclei are colored blue, and the cytoplasm of apoptotic caspase-3^+^/7^+^ cells is colored green. *—significant difference compared to the apoptosis rate of negative control cells. (D) Flow cytometry revealed no changes in apoptosis of SK-MEL-1 cells after miR-4286 inhibition. (E) The percentage of viable, early apoptotic, late apoptotic/necrotic SK-MEL-1 cells. (F) Fluorescent microscopy of alive and apoptotic SK-MEL-1 cells: viable cell nuclei are colored blue, and the cytoplasm of caspase-3^+^/7^+^ apoptotic cells is colored green.

To further investigate the effect of miR-4286 on apoptosis in cancer cells we transfected cells with the miR-4286 inhibitor and examined its effect on melanoma cells. Flow cytometry showed that miR-4286 inhibition in BRO melanoma cells increased the apoptosis rate 2.6-fold and the number of melanoma cells in the pre-apoptotic phase also increased 2-fold (Fig 5, S7 Table). A fluorescence microscopy assay revealed increase in the caspase 3/7 activity in miR-4286-inhibitor transfected cells.

#### Migration, invasion analysis

**Fig 6:**
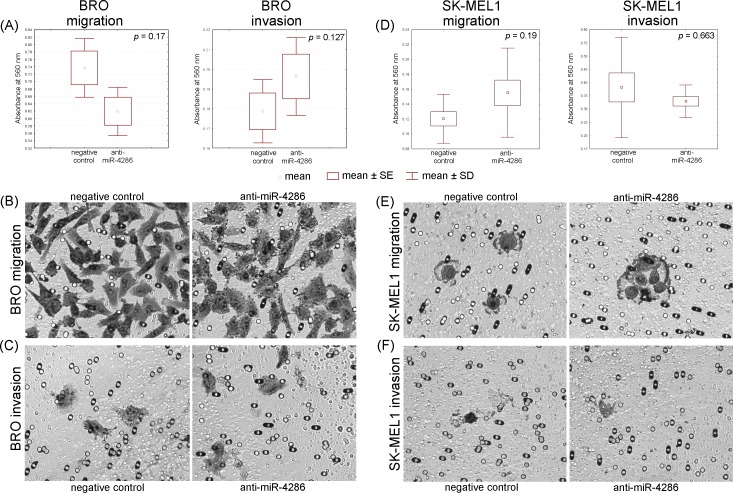
Migration and invasion assays of melanoma cells BRO and SK-MEL1 with miR-4286 inhibition. Anti-miR-4286 does not influence these processes in BRO and SK-MEL-1 cells. (A) Migration and invasion graphs of BRO melanoma cells. Microscopy of polycarbonate membrane with migrated (B) and invaded (C) BRO melanoma cells (matrigel was removed before staining in accordance with manufacturer’s protocol). (D) Migration and invasion graphs of SK-MEL-1 cells. Microscopy of polycarbonate membrane with migrated (E) and invaded (F) cells SK-MEL-1.

We performed an *in vitro* cell invasion and migration assay to further determine if miR-4286 affects cell invasion and migration. The inhibition of miR-4286 did not alter melanoma cell migration and invasion as shown in Fig 6 and S8 Table. These results suggest that the down-regulation of miR-4286 does not inhibit the migratory and invasive capacities of melanoma cells *in vitro*.

#### MiR-4286 gene targets validation by real-time PCR after miR-4286 inhibitor application

**Fig 7:**
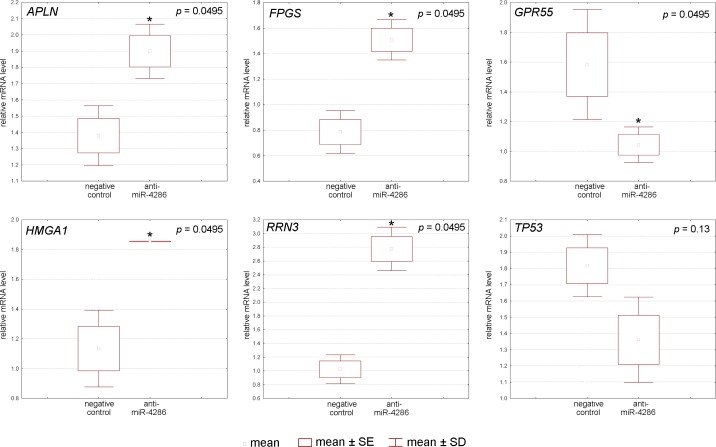
Relative expression of miR-4286 gene targets after specific miR-4286 inhibitor application to BRO melanoma cells.

**Fig 8:**
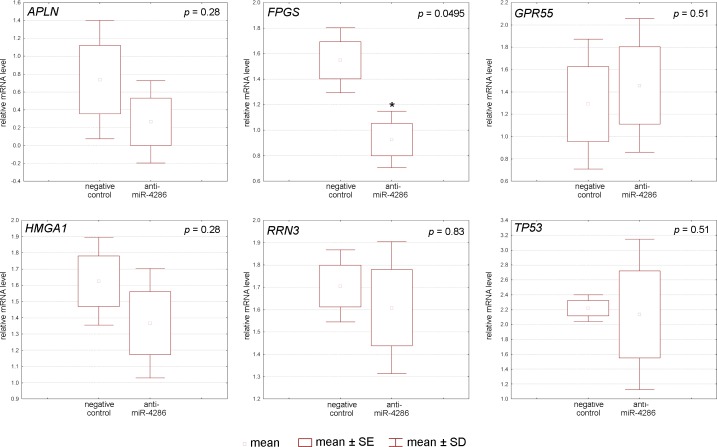
Relative expression of miR-4286 gene targets after specific miR-4286 inhibitor application to SK-MEL-1 melanoma cells.

To understand the mechanism by which miR-4286 affects cell proliferation and apoptosis, we studied the expression levels of miR-4286 target genes that regulate these processes. Indeed, miR-4286 inhibition resulted in degradation of folylpolyglutamate synthase, RNA polymerase I-specific transcription initiation factor, apelin, G-protein-coupled receptor 55, and high-mobility group A1 protein mRNA in BRO melanoma cells, as measured by real-time PCR (Fig 7, S9 Table). In SK-MEL-1 cells, only the expression of folylpolyglutamate synthase was down-regulated (Fig 8, S9 Table).

## Discussion

### MicroRNA in melanoma versus melanocytic nevi

Several studies have attempted to reveal differences in the miRNA expression profile between melanoma and benign melanocytic tumor tissues. The data reported herein are partially consistent with previously reported findings. Specifically, we found that miR-211 [29; 30; 31], miR-204 [28; 29], miR-23b-3p [28; 29], miR-26a-5p [29; 32], and miR-146a-5p [29] were down-regulated, whereas miR-155-5p was up-regulated [13; 28; 33], as also described earlier.

The present study identified several novel microRNAs aberrantly expressed in melanoma compared to melanocytic nevi. Specifically, the investigation of FFPE melanocytic tumors showed that miR-4454 and miR-7975, whose functions have not yet been studied, were overexpressed.

A pathway analysis revealed that fatty acid biosynthesis, elongation, degradation, and metabolism were altered in melanoma samples compared to nevi samples. The previous studies showed that the fatty acids play a role in melanomagenesis. In contrast to most normal cells, fatty acid synthase is overexpressed in a variety of human cancers, including cutaneous melanoma, in which its elevated expression levels are associated with tumor invasion and poor prognosis. Furthermore, fatty acid synthase inhibition induced apoptosis in melanoma cells [34]. Other aberrantly activated pathways in melanoma compared to melanocytic nevi include the AMP-activated protein kinase (AMPK), hippo and transforming growth factor-beta (TGF-beta) signaling pathways. AMPK is known as an important regulator of cellular energy homeostasis and has been shown to control tumor progression by regulating the cell cycle, protein synthesis, cell growth and survival [35].

Hippo signaling was first described to suppress proliferation and induce apoptosis. Moreover, the Hippo signaling pathway is also responsible for cell-cell adhesion interactions during metastasis development, and it has been also described to play a role in cancer stem cell regulation [36]. TGF-beta is a pleiotropic regulator of cancer cell activity that affects cell proliferation, apoptosis, differentiation, and immune function [37]. Specifically, TGF-beta induces epithelial-to-mesenchymal-like transitions in melanoma cells and peritumoral angiogenesis [38; 39]; recent studies by Fedorenko et al. showed that treating melanoma cells with vemurafenib induce fibroblasts activity by releasing TGF-beta, which leads to therapeutic resistance [40].

### Impact of miR-4286 on melanoma cells behavior

The inhibition of miR-4286 led to diminishing cell proliferation rate in both melanoma cell lines and triggered an increase in BRO apoptotic melanoma cells as indicated by elevated numbers of Annexin V+/7AAD- detected by flow cytometry. Unfortunately, Annexin V/7AAD staining does not allow to clearly differentiate late apoptosis and necrosis. Our data show that inhibition of miR-4286 increases the apoptosis and necrosis of BRO melanoma cells that is partially referable to the apoptosis induction. Moreover, the inhibition of miR-4286 down-regulated the expression of its targets. Taken together, these findings suggest that miR-4286 is involved in cell proliferation and apoptosis regulation. Apelin modulates these processes via PI3/Akt signaling pathway [41, 42], and G protein-coupled receptor 55 triggers MAPK cascade [43]. Furthermore, high-mobility group A1 protein is involved in Wnt/β-catenin pathway [44]. SK-MEL-1 melanoma cells proliferation was altered after miR-4286 inhibitor application, which matched the changes in the levels of folylpolyglutamate synthase (FPGS), an enzyme crucial for survival of dividing cells. FPGS expression levels showed different tendencies in BRO and SK-MEL-1 cells. This phenomenon can be explained by different action of FPGS in melanoma cells due to its oxidative status. Melanoma metastases of visceral organs (BRO melanoma cells) are more subjected to oxidative stress that leads to FPGS activation as folate metabolism is a major source of NADPH for oxidative stress reactions. On the contrary, primary tumors and regional lymph nodes metastasis (SK-MEL-1) showed a reduced expression of folate metabolism enzymes because oxidative stress reactions are not present [45]. MiR-4286 does not target p53 since its levels were not altered after miR-4286 inhibitor application that may be due to the activation of p53-independent apoptosis in cells.

## Conclusion

The obtained data demonstrate that miR-4286 contributes to the regulation of melanoma cell proliferation and apoptosis by triggering several pathways, suggesting that it exerts a pleotropic effect.

## Supporting Information

S1 Table Differentially expressed microRNA in melanoma tissues compared to melanocytic nevi according to a microarray analysis. (XLSX)

S2 Table Expression levels of microRNAs in melanocytic nevi and melanoma according to a real-time PCR analysis. (DOCX)

S3 Table Relative expression levels of miR-4286 in melanoma cell lines after miR-4286 inhibition. (DOCX)

S4 Table Signaling pathways associated with altered microRNA levels in melanoma tissues compared to melanocytic nevi. (DOCX)

S5 Table Target genes of miR-4286 and their biological function. (DOCX)

S6 Table Results of the MTT-test. (DOCX)

S7 Table Results of the apoptosis assay evaluated by flow cytometry. (DOCX)

S8 Table Results of the migration and invasion study. (DOCX)

S9 Table Expression levels of miR-4286 target genes in melanoma cell lines after miR-4286 inhibition. (DOCX)
