# Supplementary material for: Antiproliferative and Pro-Apoptotic Effects of MiR-4286 Inhibition in Melanoma Cells
Source: PLoS One. 2016 Dec 22;11(12):e0168229. doi: 10.1371/journal.pone.0168229 (PMC5179095; doi:10.1371/journal.pone.0168229)
Supplement: S2 Table — (DOCX) [file pone.0168229.s002.docx]

Table S2. Expression levels of microRNAs in melanocytic nevi and melanoma according to a real-time PCR analysis. The data correspond to the graphs in Fig. 2.

| microRNA | Relative Quantity in nevi,  median [25%; 75%] | Relative Quantity in melanoma,  median [25%; 75%] | P |
| --- | --- | --- | --- |
| miR-363-3p | 0.000055 [0.000346; 0.000167] | 0.002113 [0.000071; 0.011204] | 0.23 |
| miR-513a-5p | 0.002070 [0.001279; 0.002521] | 0.000000 [0.000000; 0.000514] | 0.07 |
| miR-3591-3p | 0.000482 [0.000256; 0.002920] | 0.000000 [0.000000; 0.000030] | 0.04 |
