# Supplementary material for: Antiproliferative and Pro-Apoptotic Effects of MiR-4286 Inhibition in Melanoma Cells
Source: PLoS One. 2016 Dec 22;11(12):e0168229. doi: 10.1371/journal.pone.0168229 (PMC5179095; doi:10.1371/journal.pone.0168229)
Supplement: S3 Table — (DOCX) [file pone.0168229.s003.docx]

Table S3. Relative expression levels of miR-4286 in melanoma cell lines after miR-4286 inhibition (normalized by the geometric mean of SnoRNU6 and RNU6B expression levels). The data correspond to the graphs in Fig. 3

| Cell line | Relative quantitation (RQ),  mean ± SEM | | P |
| --- | --- | --- | --- |
|  | Negative control | Anti-miR-4286 |  |
| BRO | 5.236992±1.467341 | 0.105141±0.046421 | 0.0495 |
| SK-MEL1 | 0,86859±0,117729 | 0,144563±0,023601 | 0.0495 |
