# Supplementary material for: Antiproliferative and Pro-Apoptotic Effects of MiR-4286 Inhibition in Melanoma Cells
Source: PLoS One. 2016 Dec 22;11(12):e0168229. doi: 10.1371/journal.pone.0168229 (PMC5179095; doi:10.1371/journal.pone.0168229)
Supplement: S4 Table — (DOCX) [file pone.0168229.s004.docx]

Table S4. Signaling pathways associated with altered microRNA levels in melanoma tissues compared to melanocytic nevi

| **№** | **Signaling pathway** |
| --- | --- |
| 1 | Adherens junction |
| 2 | Alzheimer's disease |
| 3 | AMPK signaling pathway |
| 4 | Arrhythmogenic right ventricular cardiomyopathy (ARVC) |
| 5 | Bacterial invasion of epithelial cells |
| 6 | Bladder cancer |
| 7 | Cell cycle |
| 8 | Chronic myeloid leukemia |
| 9 | Colorectal cancer |
| 10 | Endocytosis |
| 11 | Endometrial cancer |
| 12 | Estrogen signaling pathway |
| 13 | Fatty acid biosynthesis |
| 14 | Fatty acid degradation |
| 15 | Fatty acid elongation |
| 16 | Fatty acid metabolism |
| 17 | Focal adhesion |
| 18 | FoxO signaling pathway |
| 19 | Glioma |
| 20 | Glycosaminoglycan biosynthesis - keratan sulfate |
| 21 | Hepatitis B |
| 22 | Hippo signaling pathway |
| 23 | Huntington's disease |
| 24 | Insulin signaling pathway |
| 25 | Lysine degradation |
| 26 | Melanoma |
| 27 | Mismatch repair |
| 28 | mRNA surveillance pathway |
| 29 | Neurotrophin signaling pathway |
| 30 | N-Glycan biosynthesis |
| 31 | Non-alcoholic fatty liver disease (NAFLD) |
| 32 | Non-small cell lung cancer |
| 33 | Oocyte meiosis |
| 34 | Other types of O-glycan biosynthesis |
| 35 | p53 signaling pathway |
| 36 | Pancreatic cancer |
| 37 | Pantothenate and CoA biosynthesis |
| 38 | Prion diseases |
| 39 | Progesterone-mediated oocyte maturation |
| 40 | Prostate cancer |
| 41 | Protein processing in endoplasmic reticulum |
| 42 | Regulation of actin cytoskeleton |
| 43 | Ribosome |
| 44 | RNA degradation |
| 45 | RNA transport |
| 46 | Signaling pathways regulating pluripotency of stem cells |
| 47 | Sphingolipid signaling pathway |
| 48 | Spliceosome |
| 49 | Steroid biosynthesis |
| 50 | TGF-beta signaling pathway |
| 51 | Ubiquitin mediated proteolysis |
| 52 | Viral carcinogenesis |
