# Supplementary material for: Antiproliferative and Pro-Apoptotic Effects of MiR-4286 Inhibition in Melanoma Cells
Source: PLoS One. 2016 Dec 22;11(12):e0168229. doi: 10.1371/journal.pone.0168229 (PMC5179095; doi:10.1371/journal.pone.0168229)
Supplement: S5 Table — (DOCX) [file pone.0168229.s005.docx]

Table S5. Target genes of miR-4286 and their biological function

| № | **Target gene** | **Biological processes involved** |
| --- | --- | --- |
| 1. | ACBD7 | [fatty-acyl-CoA binding](http://amigo.geneontology.org/amigo/term/GO:0000062), [lipid binding](http://amigo.geneontology.org/amigo/term/GO:0008289) |
| 2. | ADRA2B | [G-protein coupled receptor signaling pathway](http://amigo.geneontology.org/amigo/term/GO:0007186), [activation of MAPK activity by adrenergic receptor signaling pathway](http://amigo.geneontology.org/amigo/term/GO:0071883), [activation of protein kinase B activity](http://amigo.geneontology.org/amigo/term/GO:0032148), [cell-cell signaling](http://amigo.geneontology.org/amigo/term/GO:0007267), [epidermal growth factor-activated receptor transactivation by G-protein coupled receptor signaling pathway](http://amigo.geneontology.org/amigo/term/GO:0035625), [positive regulation of neuron differentiation](http://amigo.geneontology.org/amigo/term/GO:0045666) |
| 3. | AHSA2 | [positive regulation of ATPase activity](http://amigo.geneontology.org/amigo/term/GO:0032781) |
| 4. | ANKFY1 | [endosomal vesicle fusion](http://amigo.geneontology.org/amigo/term/GO:0034058), [positive regulation of pinocytosis](http://amigo.geneontology.org/amigo/term/GO:0048549) |
| 5. | APLN | [immune response](http://amigo.geneontology.org/amigo/term/GO:0006955), [positive regulation of cell proliferation](http://amigo.geneontology.org/amigo/term/GO:0008284), [negative regulation of vasoconstriction](http://amigo.geneontology.org/amigo/term/GO:0045906), [positive regulation of vasodilation](http://amigo.geneontology.org/amigo/term/GO:0045909), [signal transduction](http://amigo.geneontology.org/amigo/term/GO:0007165) |
| 6. | APOL6 | [lipoprotein metabolic process](http://amigo.geneontology.org/amigo/term/GO:0042157), [lipid transport](http://amigo.geneontology.org/amigo/term/GO:0006869) |
| 7. | ASB16 | [intracellular signal transduction](http://amigo.geneontology.org/amigo/term/GO:0035556), [protein ubiquitination](http://amigo.geneontology.org/amigo/term/GO:0016567) |
| 8. | ATG2A | [autophagosome assembly](http://amigo.geneontology.org/amigo/term/GO:0000045), [mitophagy](http://amigo.geneontology.org/amigo/term/GO:0000422) |
| 9. | ATP13A4 | [cation transmembrane transport](http://amigo.geneontology.org/amigo/term/GO:0098655) |
| 10. | ATP1A3 | [cellular potassium ion homeostasis](http://amigo.geneontology.org/amigo/term/GO:0030007), [cellular response to steroid hormone stimulus](http://amigo.geneontology.org/amigo/term/GO:0071383), [establishment or maintenance of transmembrane electrochemical gradient](http://amigo.geneontology.org/amigo/term/GO:0010248), [potassium ion import](http://amigo.geneontology.org/amigo/term/GO:0010107), [sodium ion export from cell](http://amigo.geneontology.org/amigo/term/GO:0036376) |
| 11. | BBS5 | [cilium assembly](http://amigo.geneontology.org/amigo/term/GO:0042384) |
| 12. | C2orf15 | [poly(A) RNA binding](http://amigo.geneontology.org/amigo/term/GO:0044822) |
| 13. | CACNA1E | [calcium ion transmembrane transport](http://amigo.geneontology.org/amigo/term/GO:0070588) |
| 14. | CCBE1 | [lymphangiogenesis](http://amigo.geneontology.org/amigo/term/GO:0001946), [positive regulation of protein processing](http://amigo.geneontology.org/amigo/term/GO:0010954), [positive regulation of vascular endothelial growth factor production](http://amigo.geneontology.org/amigo/term/GO:0010575), [positive regulation of vascular endothelial growth factor signaling pathway](http://amigo.geneontology.org/amigo/term/GO:1900748) |
| 15. | CDCA4 | [protein binding](http://amigo.geneontology.org/amigo/term/GO:0005515) |
| 16. | CLDN1 | [cell-cell junction organization](http://amigo.geneontology.org/amigo/term/GO:0045216), [bicellular tight junction assembly](http://amigo.geneontology.org/amigo/term/GO:0070830) |
| 17. | CLEC2D | [cell surface receptor signaling pathway](http://amigo.geneontology.org/amigo/term/GO:0007166) |
| 18. | CLIP1 | [microtubule bundle formation](http://amigo.geneontology.org/amigo/term/GO:0001578), [mitotic nuclear division](http://amigo.geneontology.org/amigo/term/GO:0007067) |
| 19. | CYYR1 | N/D |
| 20. | DCTN5 | [cellular protein metabolic process](http://amigo.geneontology.org/amigo/term/GO:0044267), [ER to Golgi vesicle-mediated transport](http://amigo.geneontology.org/amigo/term/GO:0006888), [antigen processing and presentation of exogenous peptide antigen via MHC class II](http://amigo.geneontology.org/amigo/term/GO:0019886) |
| 21. | DIRAS1 | [positive regulation of MAP kinase activity](http://amigo.geneontology.org/amigo/term/GO:0043406) |
| 22. | EPB41L1 | [cortical actin cytoskeleton organization](http://amigo.geneontology.org/amigo/term/GO:0030866), [synaptic transmission](http://amigo.geneontology.org/amigo/term/GO:0007268) |
| 23. | FBXO18 | [DNA duplex unwinding](http://amigo.geneontology.org/amigo/term/GO:0032508), [cell death](http://amigo.geneontology.org/amigo/term/GO:0008219), [positive regulation of intrinsic apoptotic signaling pathway in response to DNA damage](http://amigo.geneontology.org/amigo/term/GO:1902231), [protein ubiquitination](http://amigo.geneontology.org/amigo/term/GO:0016567) |
| 24. | FCGR3B | [IgG binding](http://amigo.geneontology.org/amigo/term/GO:0019864) |
| 25. | FPGS | [cell proliferation](http://amigo.geneontology.org/amigo/term/GO:0008283), [folic acid-containing compound metabolic process](http://amigo.geneontology.org/amigo/term/GO:0006760), [tetrahydrofolylpolyglutamate biosynthetic process](http://amigo.geneontology.org/amigo/term/GO:0046901), [ATP binding](http://amigo.geneontology.org/amigo/term/GO:0005524) |
| 26. | FTSJ2 | [cell proliferation](http://amigo.geneontology.org/amigo/term/GO:0008283), [rRNA methylation](http://amigo.geneontology.org/amigo/term/GO:0031167), [rRNA processing](http://amigo.geneontology.org/amigo/term/GO:0006364) |
| 27. | FURIN | [cell proliferation](http://amigo.geneontology.org/amigo/term/GO:0008283), [extracellular matrix disassembly](http://amigo.geneontology.org/amigo/term/GO:0022617), [negative regulation of transforming growth factor beta1 production](http://amigo.geneontology.org/amigo/term/GO:0032911), [negative regulation of low-density lipoprotein particle receptor catabolic process](http://amigo.geneontology.org/amigo/term/GO:0032804), [peptide hormone processing](http://amigo.geneontology.org/amigo/term/GO:0016486), [protein processing](http://amigo.geneontology.org/amigo/term/GO:0016485) |
| 28. | GATS | N/D |
| 29. | GOSR1 | [SNAP receptor activity](http://amigo.geneontology.org/amigo/term/GO:0005484), [SNARE binding](http://amigo.geneontology.org/amigo/term/GO:0000149) |
| 30. | GPR55 | [positive regulation of ERK1 and ERK2 cascade](http://amigo.geneontology.org/amigo/term/GO:0070374), [positive regulation of Rho protein signal transduction](http://amigo.geneontology.org/amigo/term/GO:0035025), [cannabinoid signaling pathway](http://amigo.geneontology.org/amigo/term/GO:0038171) |
| 31. | GPS1 | [JNK cascade](http://amigo.geneontology.org/amigo/term/GO:0007254), [inactivation of MAPK activity](http://amigo.geneontology.org/amigo/term/GO:0000188), [negative regulation of GTPase activity](http://amigo.geneontology.org/amigo/term/GO:0034260) |
| 32. | HMGA1 | [DNA unwinding involved in DNA replication](http://amigo.geneontology.org/amigo/term/GO:0006268), [base-excision repair](http://amigo.geneontology.org/amigo/term/GO:0006284), [negative regulation of cell proliferation](http://amigo.geneontology.org/amigo/term/GO:0008285), [negative regulation of chromatin silencing](http://amigo.geneontology.org/amigo/term/GO:0031936), [negative regulation of transcription, DNA-templated](http://amigo.geneontology.org/amigo/term/GO:0045892), [nucleosome disassembly](http://amigo.geneontology.org/amigo/term/GO:0006337), [oncogene-induced cell senescence](http://amigo.geneontology.org/amigo/term/GO:0090402), [positive regulation of cellular senescence](http://amigo.geneontology.org/amigo/term/GO:2000774), [positive regulation of transcription, DNA-templated](http://amigo.geneontology.org/amigo/term/GO:0045893), [protein complex assembly](http://amigo.geneontology.org/amigo/term/GO:0006461), [regulation of transcription, DNA-templated](http://amigo.geneontology.org/amigo/term/GO:0006355), [senescence-associated heterochromatin focus assembly](http://amigo.geneontology.org/amigo/term/GO:0035986) |
| 33. | HOXA13 | [DNA binding](http://amigo.geneontology.org/amigo/term/GO:0003677) |
| 34. | HSPA4 | [chaperone-mediated protein complex assembly](http://amigo.geneontology.org/amigo/term/GO:0051131), [protein import into mitochondrial outer membrane](http://amigo.geneontology.org/amigo/term/GO:0045040) |
| 35. | IP6K1 | [phosphatidylinositolphosphorylation](http://amigo.geneontology.org/amigo/term/GO:0046854) |
| 36. | KHSRP | [RNA splicing](http://amigo.geneontology.org/amigo/term/GO:0008380) |
| 37. | KIAA1217 | [embryonic skeletal system development](http://amigo.geneontology.org/amigo/term/GO:0048706) |
| 38. | KLHDC3 | [reciprocal meiotic recombination](http://amigo.geneontology.org/amigo/term/GO:0007131) |
| 39. | KSR1 | [MAPK cascade](http://amigo.geneontology.org/amigo/term/GO:0000165), [Ras protein signal transduction](http://amigo.geneontology.org/amigo/term/GO:0007265), [epidermal growth factor receptor signaling pathway](http://amigo.geneontology.org/amigo/term/GO:0007173), [fibroblast growth factor receptor signaling pathway](http://amigo.geneontology.org/amigo/term/GO:0008543), [vascular endothelial growth factor receptor signaling pathway](http://amigo.geneontology.org/amigo/term/GO:0048010) |
| 40. | LDLR | [cholesterol homeostasis](http://amigo.geneontology.org/amigo/term/GO:0042632), [cholesterol transport](http://amigo.geneontology.org/amigo/term/GO:0030301), [endocytosis](http://amigo.geneontology.org/amigo/term/GO:0006897), fat-soluble vitamin metabolic process, [intestinal cholesterol absorption](http://amigo.geneontology.org/amigo/term/GO:0030299), [lipid metabolic process](http://amigo.geneontology.org/amigo/term/GO:0006629), [lipoprotein metabolic process](http://amigo.geneontology.org/amigo/term/GO:0042157), [positive regulation of triglyceride biosynthetic process](http://amigo.geneontology.org/amigo/term/GO:0010867), [retinoid metabolic process](http://amigo.geneontology.org/amigo/term/GO:0001523) |
| 41. | LYRM4 | [small molecule metabolic process](http://amigo.geneontology.org/amigo/term/GO:0044281) |
| 42. | MAN1A2 | [calcium ion binding](http://amigo.geneontology.org/amigo/term/GO:0005509), [N-glycan processing](http://amigo.geneontology.org/amigo/term/GO:0006491) |
| 43. | MAPK1 | [MAPK cascade](http://amigo.geneontology.org/amigo/term/GO:0000165), [activation of MAPK activity](http://amigo.geneontology.org/amigo/term/GO:0000187), [apoptotic process](http://amigo.geneontology.org/amigo/term/GO:0006915), [cell cycle](http://amigo.geneontology.org/amigo/term/GO:0007049), [ERBB signaling pathway](http://amigo.geneontology.org/amigo/term/GO:0038127),[ERK1 and ERK2 cascade](http://amigo.geneontology.org/amigo/term/GO:0070371), [T cell receptor signaling pathway](http://amigo.geneontology.org/amigo/term/GO:0050852) |
| 44. | MKNK2 | [cell surface receptor signaling pathway](http://amigo.geneontology.org/amigo/term/GO:0007166), [cellular response to arsenic-containing substance](http://amigo.geneontology.org/amigo/term/GO:0071243), [hemopoiesis](http://amigo.geneontology.org/amigo/term/GO:0030097), [intracellular signal transduction](http://amigo.geneontology.org/amigo/term/GO:0035556), [protein phosphorylation](http://amigo.geneontology.org/amigo/term/GO:0006468) |
| 45. | MRPL30 | [mitochondrial translation](http://amigo.geneontology.org/amigo/term/GO:0032543) |
| 46. | NFYA | [regulation of transcription, DNA-templated](http://amigo.geneontology.org/amigo/term/GO:0006355), [transcription from RNA polymerase II promoter](http://amigo.geneontology.org/amigo/term/GO:0006366) |
| 47. | NOM1 | [RNA binding](http://amigo.geneontology.org/amigo/term/GO:0003723) |
| 48. | NSD1 | [positive regulation of transcription, DNA-templated](http://amigo.geneontology.org/amigo/term/GO:0045893), [regulation of RNA polymerase II regulatory region sequence-specific DNA binding](http://amigo.geneontology.org/amigo/term/GO:1903025), [regulation of histone H3-K36 methylation](http://amigo.geneontology.org/amigo/term/GO:0000414) |
| 49. | NUDT5 | [D-ribose catabolic process](http://amigo.geneontology.org/amigo/term/GO:0019303), [ribonucleoside diphosphate catabolic process](http://amigo.geneontology.org/amigo/term/GO:0009191) |
| 50. | PEX16 | [ER-dependent peroxisome organization](http://amigo.geneontology.org/amigo/term/GO:0032581), [protein localization to endoplasmic reticulum](http://amigo.geneontology.org/amigo/term/GO:0070972), [protein to membrane docking](http://amigo.geneontology.org/amigo/term/GO:0022615) |
| 51. | PGK1 | [epithelial cell differentiation](http://amigo.geneontology.org/amigo/term/GO:0030855), [gluconeogenesis](http://amigo.geneontology.org/amigo/term/GO:0006094), [glucose metabolic process](http://amigo.geneontology.org/amigo/term/GO:0006006) |
| 52. | PGPEP1 | [proteolysis](http://amigo.geneontology.org/amigo/term/GO:0006508) |
| 53. | PIK3C2B | [cell migration](http://amigo.geneontology.org/amigo/term/GO:0016477), [protein kinase B signaling](http://amigo.geneontology.org/amigo/term/GO:0043491), [phosphatidylinositol-3-phosphate biosynthetic process](http://amigo.geneontology.org/amigo/term/GO:0036092) |
| 54. | PQLC1 | [integral component of membrane](http://amigo.geneontology.org/amigo/term/GO:0016021) |
| 55. | PRKAG1 | [fatty acid biosynthetic process](http://amigo.geneontology.org/amigo/term/GO:0006633), [positive regulation of gene expression](http://amigo.geneontology.org/amigo/term/GO:0010628), [macroautophagy](http://amigo.geneontology.org/amigo/term/GO:0016236), [signal transduction](http://amigo.geneontology.org/amigo/term/GO:0007165), [regulation of glycolytic process](http://amigo.geneontology.org/amigo/term/GO:0006110), [insulin receptor signaling pathway](http://amigo.geneontology.org/amigo/term/GO:0008286) |
| 56. | PRR15 | [multicellular organism development](http://amigo.geneontology.org/amigo/term/GO:0007275) |
| 57. | PSMG1 | [proteasome assembly](http://amigo.geneontology.org/amigo/term/GO:0043248) |
| 58. | R3HDM4 | [nucleic acid binding](http://amigo.geneontology.org/amigo/term/GO:0003676) |
| 59. | RABGAP1 | [cell cycle](http://amigo.geneontology.org/amigo/term/GO:0007049), [positive regulation of GTPase activity](http://amigo.geneontology.org/amigo/term/GO:0043547) |
| 60. | RAPGEF3 | [angiogenesis](http://amigo.geneontology.org/amigo/term/GO:0001525), [cell proliferation](http://amigo.geneontology.org/amigo/term/GO:0008283), [cellular response to transforming growth factor beta stimulus](http://amigo.geneontology.org/amigo/term/GO:0071560), [negative regulation of collagen biosynthetic process](http://amigo.geneontology.org/amigo/term/GO:0032966), [positive regulation of GTPase activity](http://amigo.geneontology.org/amigo/term/GO:0043547), [positive regulation of angiogenesis](http://amigo.geneontology.org/amigo/term/GO:0045766), [positive regulation of calcium ion transmembrane transport](http://amigo.geneontology.org/amigo/term/GO:1904427) |
| 61. | REL | [negative regulation of gene expression](http://amigo.geneontology.org/amigo/term/GO:0010629), [negative regulation of interferon-beta production](http://amigo.geneontology.org/amigo/term/GO:0032688), [positive regulation of I-kappa B kinase/NF-kappaB signaling](http://amigo.geneontology.org/amigo/term/GO:0043123), [response to cytokine](http://amigo.geneontology.org/amigo/term/GO:0034097) |
| 62. | RGP1 | [negative regulation of cellular protein catabolic process](http://amigo.geneontology.org/amigo/term/GO:1903363), [positive regulation of GTPase activity](http://amigo.geneontology.org/amigo/term/GO:0043547) |
| 63. | RIMS3 | [ion channel binding](http://amigo.geneontology.org/amigo/term/GO:0044325) |
| 64. | RPS6 | [T cell proliferation involved in immune response](http://amigo.geneontology.org/amigo/term/GO:0002309), [TOR signaling](http://amigo.geneontology.org/amigo/term/GO:0031929), [activation-induced cell death of T cells](http://amigo.geneontology.org/amigo/term/GO:0006924),[glucose homeostasis](http://amigo.geneontology.org/amigo/term/GO:0042593), [positive regulation of apoptotic process](http://amigo.geneontology.org/amigo/term/GO:0043065) |
| 65. | RRN3 | [cell proliferation](http://amigo.geneontology.org/amigo/term/GO:0008283), [homeostasis of number of cells](http://amigo.geneontology.org/amigo/term/GO:0048872),[negative regulation of intrinsic apoptotic signaling pathway by p53 class mediator](http://amigo.geneontology.org/amigo/term/GO:1902254) |
| 66. | SCAI | [negative regulation of cell migration](http://amigo.geneontology.org/amigo/term/GO:0030336), [negative regulation of nucleic acid-templated transcription](http://amigo.geneontology.org/amigo/term/GO:1903507) |
| 67. | SCNN1G | [ion channel activity](http://amigo.geneontology.org/amigo/term/GO:0005216), [multicellular organismal water homeostasis](http://amigo.geneontology.org/amigo/term/GO:0050891), [sodium ion transmembrane transport](http://amigo.geneontology.org/amigo/term/GO:0035725) |
| 68. | SEC61A2 | [antigen processing and presentation of peptide antigen via MHC class I](http://amigo.geneontology.org/amigo/term/GO:0002474) |
| 69. | SEMA4D | [cell adhesion](http://amigo.geneontology.org/amigo/term/GO:0007155), [immune response](http://amigo.geneontology.org/amigo/term/GO:0006955), [leukocyte aggregation](http://amigo.geneontology.org/amigo/term/GO:0070486), [negative regulation of apoptotic process](http://amigo.geneontology.org/amigo/term/GO:0043066), [negative regulation of cell adhesion](http://amigo.geneontology.org/amigo/term/GO:0007162), [negative regulation of transcription from RNA polymerase II promoter](http://amigo.geneontology.org/amigo/term/GO:0000122), [positive regulation of GTPase activity](http://amigo.geneontology.org/amigo/term/GO:0043547), [regulation of cell shape](http://amigo.geneontology.org/amigo/term/GO:0008360) |
| 70. | SIRPA | [cell adhesion](http://amigo.geneontology.org/amigo/term/GO:0007155), [leukocyte migration](http://amigo.geneontology.org/amigo/term/GO:0050900) |
| 71. | SLC39A13 | [zinc ion transmembrane transporter activity](http://amigo.geneontology.org/amigo/term/GO:0005385) |
| 72. | SLC5A2 | [carbohydrate metabolic process](http://amigo.geneontology.org/amigo/term/GO:0005975), [glucose transmembrane transport](http://amigo.geneontology.org/amigo/term/GO:1904659), [sodium ion transport](http://amigo.geneontology.org/amigo/term/GO:0006814) |
| 73. | SLC6A17 | [amino acid transmembrane transporter activity](http://amigo.geneontology.org/amigo/term/GO:0015171) |
| 74. | SLFN5 | [cell differentiation](http://amigo.geneontology.org/amigo/term/GO:0030154) |
| 75. | SREBF1 | [cellular response to fatty acid](http://amigo.geneontology.org/amigo/term/GO:0071398), [cholesterol metabolic process](http://amigo.geneontology.org/amigo/term/GO:0008203), [circadian rhythm](http://amigo.geneontology.org/amigo/term/GO:0007623), [fat cell differentiation](http://amigo.geneontology.org/amigo/term/GO:0045444), [insulin receptor signaling pathway](http://amigo.geneontology.org/amigo/term/GO:0008286), [lipid biosynthetic process](http://amigo.geneontology.org/amigo/term/GO:0008610), [lipid metabolic process](http://amigo.geneontology.org/amigo/term/GO:0006629), [negative regulation of insulin secretion](http://amigo.geneontology.org/amigo/term/GO:0046676), [positive regulation of cholesterol biosynthetic process](http://amigo.geneontology.org/amigo/term/GO:0045542) |
| 76. | ST6GALNAC3 | [glycoprotein metabolic process](http://amigo.geneontology.org/amigo/term/GO:0009100), [glycosphingolipid metabolic process](http://amigo.geneontology.org/amigo/term/GO:0006687),[sialylation](http://amigo.geneontology.org/amigo/term/GO:0097503) |
| 77. | SUN2 | [mitotic spindle organization](http://amigo.geneontology.org/amigo/term/GO:0007052), [microtubule binding](http://amigo.geneontology.org/amigo/term/GO:0008017), [lamin binding](http://amigo.geneontology.org/amigo/term/GO:0005521) |
| 78. | TAOK1 | [MAPK cascade](http://amigo.geneontology.org/amigo/term/GO:0000165), [cellular response to DNA damage stimulus](http://amigo.geneontology.org/amigo/term/GO:0006974), [execution phase of apoptosis](http://amigo.geneontology.org/amigo/term/GO:0097194), [mitotic cell cycle](http://amigo.geneontology.org/amigo/term/GO:0000278), [positive regulation of stress-activated MAPK cascade](http://amigo.geneontology.org/amigo/term/GO:0032874), [regulation of mitotic cell cycle](http://amigo.geneontology.org/amigo/term/GO:0007346),  [spindle checkpoint](http://amigo.geneontology.org/amigo/term/GO:0031577) signaling pathway |
| 79. | TBX15 | [negative regulation of transcription from RNA polymerase II promoter](http://amigo.geneontology.org/amigo/term/GO:0000122), [transcription, DNA-templated](http://amigo.geneontology.org/amigo/term/GO:0006351) |
| 80. | TMEM109 | [negative regulation of cell death](http://amigo.geneontology.org/amigo/term/GO:0060548), [intrinsic apoptotic signaling pathway in response to DNA damage by p53 class mediator](http://amigo.geneontology.org/amigo/term/GO:0042771), [ion transport](http://amigo.geneontology.org/amigo/term/GO:0006811) |
| 81. | TMEM151B | transmembrane protein |
| 82. | TMSB4X | [actin filament organization](http://amigo.geneontology.org/amigo/term/GO:0007015), [regulation of cell migration](http://amigo.geneontology.org/amigo/term/GO:0030334) |
| 83. | TNPO3 | [splicing factor protein import into nucleus](http://amigo.geneontology.org/amigo/term/GO:0035048) |
| 84. | TP53 | [DNA damage response, signal transduction by p53 class mediator](http://amigo.geneontology.org/amigo/term/GO:0030330), [DNA repair](http://amigo.geneontology.org/amigo/term/GO:0006281), [Ras protein signal transduction](http://amigo.geneontology.org/amigo/term/GO:0007265), [apoptotic process](http://amigo.geneontology.org/amigo/term/GO:0006915), [cell aging](http://amigo.geneontology.org/amigo/term/GO:0007569), [cell cycle arrest](http://amigo.geneontology.org/amigo/term/GO:0007050), [cell differentiation](http://amigo.geneontology.org/amigo/term/GO:0030154), [cell proliferation](http://amigo.geneontology.org/amigo/term/GO:0008283), [cellular protein localization](http://amigo.geneontology.org/amigo/term/GO:0034613), [cellular response to hypoxia](http://amigo.geneontology.org/amigo/term/GO:0071456) |
| 85. | TRAF3 | [apoptotic process](http://amigo.geneontology.org/amigo/term/GO:0006915), [negative regulation of NF-kappa B transcription factor activity](http://amigo.geneontology.org/amigo/term/GO:0032088), [regulation of apoptotic process](http://amigo.geneontology.org/amigo/term/GO:0042981), [regulation of cytokine production](http://amigo.geneontology.org/amigo/term/GO:0001817), [regulation of interferon-beta production](http://amigo.geneontology.org/amigo/term/GO:0032648), [regulation of proteolysis](http://amigo.geneontology.org/amigo/term/GO:0030162), [signal transduction](http://amigo.geneontology.org/amigo/term/GO:0007165), [tumor necrosis factor-mediated signaling pathway](http://amigo.geneontology.org/amigo/term/GO:0033209) |
| 86. | TRPC4AP | [ion transmembrane transport](http://amigo.geneontology.org/amigo/term/GO:0034220), [calcium ion transmembrane transport](http://amigo.geneontology.org/amigo/term/GO:0070588), [protein ubiquitination](http://amigo.geneontology.org/amigo/term/GO:0016567), [transmembrane transport](http://amigo.geneontology.org/amigo/term/GO:0055085) |
| 87. | TSC22D2 | [regulation of transcription, DNA-templated](http://amigo.geneontology.org/amigo/term/GO:0006355), [response to osmotic stress](http://amigo.geneontology.org/amigo/term/GO:0006970) |
| 88. | UBXN11 | [proteinbinding](http://amigo.geneontology.org/amigo/term/GO:0005515), [ubiquitinbinding](http://amigo.geneontology.org/amigo/term/GO:0043130) |
| 89. | XRRA1 | [response to X-ray](http://amigo.geneontology.org/amigo/term/GO:0010165) |
| 90. | ZBTB20 | [positive regulation of interferon-beta production](http://amigo.geneontology.org/amigo/term/GO:0032728), [positive regulation of interleukin-6 production](http://amigo.geneontology.org/amigo/term/GO:0032755), [positive regulation of tumor necrosis factor production](http://amigo.geneontology.org/amigo/term/GO:0032760), [metal ion binding](http://amigo.geneontology.org/amigo/term/GO:0046872) |
| 91. | ZBTB39 | [regulation of transcription, DNA-templated](http://amigo.geneontology.org/amigo/term/GO:0006355) |
| 92. | ZBTB7A | [cell differentiation](http://amigo.geneontology.org/amigo/term/GO:0030154), [multicellular organism development](http://amigo.geneontology.org/amigo/term/GO:0007275), [negative regulation of transcription, DNA-templated](http://amigo.geneontology.org/amigo/term/GO:0045892) |
| 93. | ZFP64 | [regulation of transcription, DNA-templated](http://amigo.geneontology.org/amigo/term/GO:0006355), [transcription, DNA-templated](http://amigo.geneontology.org/amigo/term/GO:0006351) |
| 94. | ZNF35 | [cellular response to retinoic acid](http://amigo.geneontology.org/amigo/term/GO:0071300), [regulation of transcription, DNA-templated](http://amigo.geneontology.org/amigo/term/GO:0006355), [spermatogenesis](http://amigo.geneontology.org/amigo/term/GO:0007283) |
