# Supplementary material for: Antiproliferative and Pro-Apoptotic Effects of MiR-4286 Inhibition in Melanoma Cells
Source: PLoS One. 2016 Dec 22;11(12):e0168229. doi: 10.1371/journal.pone.0168229 (PMC5179095; doi:10.1371/journal.pone.0168229)
Supplement: S6 Table — (DOCX) [file pone.0168229.s006.docx]

Table S6. Results of the MTT-test. The data correspond to the graphs in Fig. 4

| Cell line | Time after transfection | Absorbance at 560 nm, mean ± SEM | | P |
| --- | --- | --- | --- | --- |
|  |  | Negative control | AntimiR-4286 |  |
| BRO | 24 h | 0.112333±0.002603 | 0.074333±0.002404 | 0.0495 |
|  | 48 h | 0.140667±0.009684 | 0.090333±0.006489 | 0.0495 |
|  | 72 h | 0.232333±0.006888 | 0.153333±0.000333 | 0.0463 |
|  | 96 h | 0.384000±0.025384 | 0.226333±0.010990 | 0.0495 |
| SK-MEL1 | 24 h | 1.241000±0.060451 | 1.181000±0.040262 | 0.2800 |
|  | 48 h | 1.474667±0.064328 | 1.606667±0.046175 | 0.2800 |
|  | 72 h | 1.862667±0.053186 | 1.776333±0.092366 | 0.5100 |
|  | 96 h | 2.224667±0.033067 | 1.608333±0.195346 | 0.0495 |
