# Supplementary material for: Antiproliferative and Pro-Apoptotic Effects of MiR-4286 Inhibition in Melanoma Cells
Source: PLoS One. 2016 Dec 22;11(12):e0168229. doi: 10.1371/journal.pone.0168229 (PMC5179095; doi:10.1371/journal.pone.0168229)
Supplement: S7 Table — (DOCX) [file pone.0168229.s007.docx]

Table S7. Results of the apoptosis assay evaluated by flow cytometry. The data correspond to the graphs in Fig. 5

| Cell line |  | Percentage, mean ± SEM | | P |
| --- | --- | --- | --- | --- |
|  |  | Negative control | AntimiR-4286 |  |
| BRO | Alive cells | 62.400000±10.33457 | 70.06667±4.80359 | 0.84 |
|  | Pre-apoptosis | 3.500000±0.230940 | 6.800000±1.738774 | 0.11 |
|  | Apoptosis | 5.766667±1.703265 | 15.23333±2.378608 | 0.0495 |
|  | Necrosis | 22.666667±8.45820 | 7.900000±0.901850 | 0.66 |
| SK-MEL1 | Alive cells | 90.433333±0.560753 | 86.566667±2.140353 | 0.38 |
|  | Pre-apoptosis | 5.533333±1.034945 | 6.633333±1.736216 | 0.66 |
|  | Apoptosis | 1.966667±0.425572 | 3.266667±0.491031 | 0.19 |
|  | Necrosis | 2.133333±0.517472 | 3.466667±0.185592 | 0.08 |
