# Supplementary material for: Antiproliferative and Pro-Apoptotic Effects of MiR-4286 Inhibition in Melanoma Cells
Source: PLoS One. 2016 Dec 22;11(12):e0168229. doi: 10.1371/journal.pone.0168229 (PMC5179095; doi:10.1371/journal.pone.0168229)
Supplement: S8 Table — (DOCX) [file pone.0168229.s008.docx]

Table S8. Results of the migration and invasion study (colorimetric format). The data correspond to the graphs in Fig. 6

| Cell line |  | Absorbance at 560 nm, means ± SEM | | P |
| --- | --- | --- | --- | --- |
|  |  | Negative control | AntimiR-4286 |  |
| BRO | Migration assay | 0.736667±0.045641 | 0.619333±0.037551 | 0.17 |
|  | Invasion assay | 0.178667±0.009207 | 0.196333±0.011348 | 0.12 |
| SK-MEL1 | Migration assay | 0.120333±0.009528 | 0.155333±0.017295 | 0.19 |
|  | Invasion assay | 0.382333±0.054725 | 0.329667±0.017817 | 0.36 |
