# Supplementary material for: Antiproliferative and Pro-Apoptotic Effects of MiR-4286 Inhibition in Melanoma Cells
Source: PLoS One. 2016 Dec 22;11(12):e0168229. doi: 10.1371/journal.pone.0168229 (PMC5179095; doi:10.1371/journal.pone.0168229)
Supplement: S9 Table — (DOCX) [file pone.0168229.s009.docx]

Table S9. Expression levels of miR-4286 target genes in melanoma cell lines after miR-4286 inhibition (normalized by the geometric mean of β-actin and HPRT-1 expression levels). The data correspond to the graphs in Fig. 7, 8

| Cell line | Target gene | Relative quantity (RQ),  mean ± SEM | | *P* |
| --- | --- | --- | --- | --- |
|  |  | Negative control | Anti-miR-4286 |  |
| BRO | APLN | 1.379364±0.106437 | 1.899728±0.095929 | 0.0495 |
|  | GPR55 | 1.583488±0.212312 | 1.044085±0.069060 | 0.0495 |
|  | HMGA1 | 1.134791±0.149028 | 1.854130±0.001822 | 0.0495 |
|  | FPGS | 0.786717±0.097157 | 1.507850±0.091347 | 0.0495 |
|  | TP53 | 1.817065±0.110366 | 1.360982±0.151231 | 0.1300 |
|  | RRN3 | 1.023991±0.120145 | 2.773556±0.182578 | 0.0495 |
| SK-MEL1 | APLN | 0.737788±0.382602 | 0.266061±0.266061 | 0.2800 |
|  | GPR55 | 1.291385±0.335397 | 1.458324±0.346018 | 0.5100 |
|  | HMGA1 | 1.625147±0.155453 | 1.366486±0.194072 | 0.2800 |
|  | FPGS | 1.547775±0.146471 | 0.927116±0.127107 | 0.0495 |
|  | TP53 | 2.220083±0.103056 | 2.136672±0.582924 | 0.5100 |
|  | RRN3 | 1.705752±0.093133 | 1.608713±0.170330 | 0.8300 |
